# Quasi-One-Dimensional van der Waals Transition Metal Trichalcogenides

**DOI:** 10.34133/research.0066

**Published:** 2023-03-14

**Authors:** Mengdi Chen, Lei Li, Manzhang Xu, Weiwei Li, Lu Zheng, Xuewen Wang

**Affiliations:** ^1^ Frontiers Science Center for Flexible Electronics (FSCFE) & Shaanxi Institute of Flexible Electronics (SIFE), Northwestern Polytechnical University (NPU), 127 West Youyi Road, Xi'an 710072, China.; ^2^ Shaanxi Key Laboratory of Flexible Electronics (KLoFE), Northwestern Polytechnical University (NPU), 127 West Youyi Road, Xi'an 710072, China.; ^3^ MIIT Key Laboratory of Flexible Electronics (KLoFE), Northwestern Polytechnical University (NPU), 127 West Youyi Road, Xi’an710072, China.; ^4^ Key Laboratory of Flexible Electronics of Zhejiang Provience, Ningbo Institute of Northwestern Polytechnical University, 218 Qingyi Road, Ningbo 315103, China.

## Abstract

The transition metal trichalcogenides (TMTCs) are quasi-one-dimensional (1D) MX3-type van der Waals layered semiconductors, where M is a transition metal element of groups IV and V, and X indicates chalcogen element. Due to the unique quasi-1D crystalline structures, they possess several novel electrical properties such as variable bandgaps, charge density waves, and superconductivity, and highly anisotropic optical, thermoelectric, and magnetic properties. The study of TMTCs plays an essential role in the 1D quantum materials field, enabling new opportunities in the material research dimension. Currently, tremendous progress in both materials and solid-state devices has been made, demonstrating promising applications in the realization of nanoelectronic devices. This review provides a comprehensive overview to survey the state of the art in materials, devices, and applications based on TMTCs. Firstly, the symbolic structure, current primary synthesis methods, and physical properties of TMTCs have been discussed. Secondly, examples of TMTC applications in various fields are presented, such as photodetectors, energy storage devices, catalysts, and sensors. Finally, we give an overview of the opportunities and future perspectives for the research of TMTCs, as well as the challenges in both basic research and practical applications.

## Introduction

The transition metal trichalcogenides (TMTCs) are commonly known as MX_3_ compounds with M = Ti, Zr, Hf, V, Nb, and Ta, and X = S, Se, and Te, which are typical quasi-one-dimensional (1D) van der Waals (vdW) structures [1–6]. The term “*quasi*” is used to differentiate it from “*true*-1D” materials [5,6]. These materials are linked by strong covalent bonds in the 1D chain direction, while weak covalent bonds are between adjacent chains. These additional bonds between the chains further arrange the 1D chains into two-dimensional (2D) sheets. Similar to other 2D structures, these sheets are stacked to form three-dimensional (3D) bulk crystals by weak vdW forces. These individual structural characteristics enable TMTCs both the advantages of 2D materials and quasi-1D properties [7–11]. It is deemed as one of the critical materials for developing the next generation of nano-electronics and has broad application prospects.

Since the early 1960s, studies related to TMTCs have been carried out, which were limited to bulk crystals [2,3,12–14]. Low-dimensional TMTCs, especially quasi-1D TMTCs, have received extensive attention in recent years because the advent of graphene (Gr) and other 2D materials has shown unprecedented material properties after reducing dimensionality. As a quasi-1D vdW material, the unique 1D chain structure of TMTCs enables its quantum properties to be expressed even in bulk crystals. Charge density wave (CDW) studies of TMTCs have been reported in the last century, such as classical NbS_3_ [15]. These 1D materials can grow directly into single-chain or few-chain structures with more substantial quantum properties [5,16–19]. Because the coherent CDW transport regime is more likely to occur in a few channels, it is predicted that when the size of the material is closer to the atomic chain, the external stimulus signal will more easily regulate the quantum properties. ZrTe_3_ and NbSe_3_ possess peculiar CDW transitions and superconductivity (SC), which can be used to fabricate radio frequency nanoelectronic devices, and also provide possibilities for information processing and quantum computing [20–26]. The asymmetric structure of TMTCs leads to the anisotropic properties in the electronic energy band, resulting in a reduction of the carrier transport dimension. The cross-sectional size of a single chain is at the atomic scale, and the sharp interface of the atoms effectively suppresses edge scattering in favor of the transport of carriers. Moreover, the TMTCs typically exhibit in-plane angle-dependent electrical, optical, thermal, and photonic properties, providing novel ideas and design freedoms for logic devices and integrated circuits [7,10,27–35]. TMTCs are promising for realizing a new generation of electronic and optoelectronic devices due to their tunable bandgap [4,7,36–41]. For instance, the bandgap of TiS_3_ will be transited from indirect to direct when the thickness was reduced to monolayer. The theoretical carrier mobility of monolayer TiS_3_ is up to 10^4^ cm^2^ V^−1^ s^−1^, which is much higher than that of monolayer MoS_2_ [39]. Additionally, TMTCs have other excellent physical properties, such as optical, thermoelectric, mechanical, and magnetic properties [16,42–57]. Therefore, several reviews have been reported to summarize the recent advances on TMTCs. However, the published reviews focus on either the crystalline structures [34] or optoelectrics properties [35] of TMTCs. A comprehensive review on the synthesis, properties, and potential applications of TMTCs is highly desirable and essential.

In this review, we systematically summarize the recent studies on TMTCs and highlight the structure, synthesis methods, extraordinary properties, and emerging applications. We start by introducing the crystal structures and electronic band structures of TMTCs, in particular their CDW and SC phases. The mainstream methods adopted to synthesize TMTCs, which include chemical vapor transport (CVT), chemical vapor deposition (CVD), atomic layer deposition (ALD), and solid-phase sulfurization method, are discussed next. We then examine the physical properties of TMTCs, including electron transport, optical, thermal, mechanically induced properties, and magnetic properties. Subsequently, potential applications such as photodetectors, energy storage devices, catalysis, and sensors are addressed. Finally, we assess the key challenges and perspectives on the future developments of TMTCs.

## Structure

### Crystal structure

**Fig. 1.**
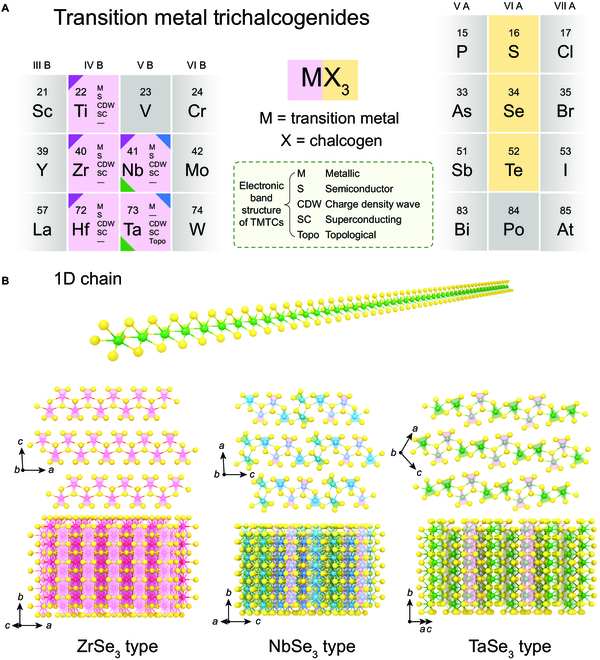
Crystal structure of TMTCs. (A) Overview of metals (highlighted in violet) and chalcogens (highlighted in yellow) that can form TMTCs in this review paper. Structural types are summarized and identified by triangles, with ZrSe_3_ in purple, NbSe_3_ in green, and TaSe_3_ in blue. The existence of the observed electron phase is also specified. (B) Atomic structure of 1D chains and typical MX_3_.

TMTCs are layered materials bound by weak vdW forces (an overview structure of TMTCs is presented in Fig. 1A). The prismatic MX_6_ chains of MX_3_ share atoms to form 1D chains as the basic structural units of each layer, where the orientation of these chains is parallel to the *b*-axis direction of the monoclinic cell, as shown in Fig. 1B [3,12,30]. Although the basic structural units of TMTCs are similar, there are some differences in the shape and assembly mode of the 1D chains. The TMTCs can be classified into 3 families with representative structures of ZrSe_3_, NbSe_3_, and TaSe_3_ [2,3,58].

The ZrSe_3_ family is a monoclinic crystal with high symmetry falling into space group *P*2_1_/*m*, which involves all the fourth group MX_3_ (M = Ti, Zr, or Hf and X = S, Se, or Te). The typical structure of the ZrSe_3_ is shown in Fig. 1B. As illustrated, all MX_3_ triangular prism chains are equivalent with an *a*/2 shift between adjacent chains. Each transition metal atom is connected to the X atom between the chain and the neighboring chain. The X atoms are combined to form a quasi-1D layered structure with anisotropic electronic and optical properties [3,12,59].

The TaSe_3_ family is another monoclinic structure crystal. Compared with the ZrSe_3_ family, each unit cell contains 4 chains, which can be divided into two types according to the length of the X–X bonds (0.258/0.291 nm) at the bottom of the triangular prism chain [3,60,61].

The triclinic structure is most stable in the NbSe_3_ family [62,63]. Compared with the ZrSe_3_ family, the NbSe_3_ family’s layer is constructed by the MX_3_ chains with highly broken symmetry. There are three nonequivalent chain structures, indicated as I, II, and III, in the NbSe_3_ family [59]. Compared with the electrons of elements in group IV, the excess electrons in the transition metal atoms of group V rupture the part of X–X bonds in the MX_3_ chains, which results in local strain. The horizontal *c*-axis is produced between the trigonal prismatic chain and the layer. The conductance and CDW can be affected by the triclinic structure [64].

The metal coordination in TMTCs is uniformly trigonometric prismatic. In addition to the combination of different chain packing arrangements within the layers described above, varying degrees of metal–metal bonding along the chain can also contribute to structural diversity [5,59]. Among the reported TMTCs, NbS_3_ has abundant polymorphisms and their crystal structure parameters are summarized in Table 1 [5,59,65–67]. So far, 7 stable experimental and forecast phases have been reported. These structures can also be attributed to the above three types. The difference lies mainly in the existence of niobium pairing in the chain.

**Table 1.** Cell parameters for reported NbS_3_ polymorphs.

Polymorph	Space group	Cell parameters						Nb–Nb distance (Å)	Ref.
		*a* (Å)	*b* (Å)	c (Å)	*α* (°)	*β* (°)	*γ* (°)		
NbS_3_-I	*P*‾1	4.963	6.730	9.144	90	97.17	90	3.045/3.702	[97]
NbS_3_-HP	*P*2_1_/*m*	9.68	3.37	14.83	90	109.9	90	3.370	[98]
NbS_3_-II	*P*2_1_/*m*	9.9	3.4	18.3	90	97	90	–	[198,199]
NbS_3_-III	–	5	–	9	90	98-99	90	–	[15]
NbS_3_-IV	*P*2_1_/*c*	6.7515	4.9736	18.1315	90	90.116	90	3.0448/3.7087	[65]
NbS_3_-V	*P*2_1_/*m*	4.950	3.358	9.079	90	97.35	90	3.358	[65]
NbS_3_-V’	–	4.828	3.346	9.113	90	81.79	90	–	[59]

### Electronic band structure

**Fig. 2.**
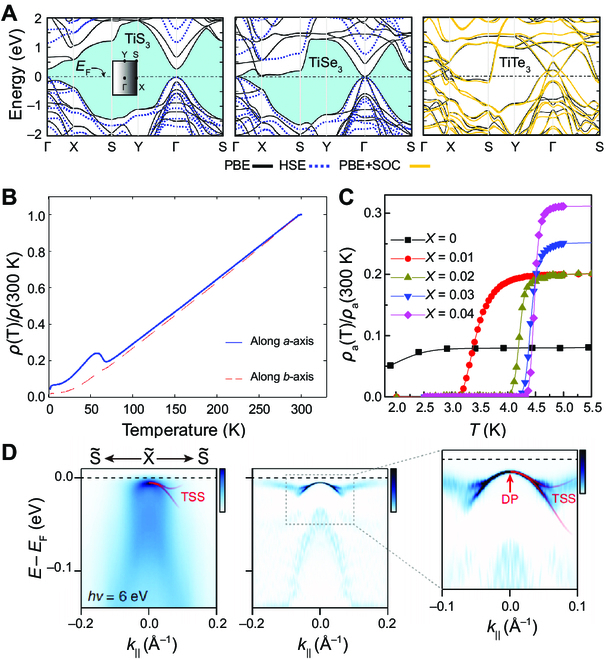
Electronic properties of TMTCs. (A) Evolution of the band structures of monolayer TiS_3_, TiSe_3,_ and TiTe_3_. The Fermi energy level is set to zero. Reproduced with permission [71]. Copyright 2017, Royal Society of Chemistry. (B) The normalized resistivity of ZrTe_3_ single crystals along the *b*-axis and *a*-axis as a function of temperature. The apparent resistivity anomaly at *T* ~ 63 K indicates the CDW transition. Reproduced with permission [88]. Copyright 2015, American Physical Society. (C) Low-temperature *ρ*_a_/*ρ*_a_(300 K) − *T* for ZrTe_3−*x*_Se*_x_*. Reproduced with permission [78]. Copyright 2016, Nature Publishing Group. (D) Band dispersion along X point to S point, measured by laser-based ARPES (hν = 6 eV). TSS, topological surface states; DP, Dirac point. Reproduced with permission [93]. Copyright 2022, American Physical Society.

The diversity of elements and crystal structures of TMTCs leads to their rich electronic properties, as summarized in Table 2. The electronic properties of the ZrSe_3_ family have been extensively studied due to the comparatively simple structure. For example, Abdulsalam and Joubert [36] calculated the energy band structure of ZrSe_3_-based TMTCs by density functional theory (DFT). The results indicated that TMTCs are all indirect bandgap semiconductors with the bandgap range of 0.44 to 2.4 eV except for antimonides and TiSe_3_ with metallic properties. NbS_3_ with polymorphism are prone to different degrees of Nb–Nb pairing along the chain due to the extra electrons provided by niobium element [5,59]. NbS_3_-I and NbS_3_-IV with Nb–Nb pairing are semiconductors. NbS_3_-V without Nb–Nb pairing has metallic properties [59,67]. The indirect bandgap of TiS_3_ will transition into direct bandgap when the thickness decreases from bulk to monolayer, which is similar to 2H-MoS_2_ [37,68 ,69]. There are two near bands with different characters and anisotropies at the valence band maximum of TiS_3_, and the order of bands can be reversed by strain engineering [70]. The monolayer TiSe_3_, ZrS_3_, and ZrSe_3_ are indirect bandgap semiconductors with a bandgap range of 0.57 to 1.90 eV, while monolayer TiTe_3_ and ZrTe_3_ are the metallic characters [37]. From the electronic band structures of TiS_3_, TiSe_3_, and TiTe_3_ (Fig. 2A), it can be seen that the bandgap is decreased with the increase of the halogen atom size. Substituting part of the halogen atoms can tailor the bandgap of TiS_3_. The monolayer TiS_3(1-*x*)_Se_3*x*_ is an excellent solar absorber material with a bandgap range of 1.2 to 1.5 eV [71]. However, a solubility limit exists in the alloy formed by element substitution, resulting in a discontinuous alloying [72]. The triple- and fewer HfTe_3_ chains confined in carbon nanotubes (CNTs) will rock distortion, driving the metal–insulator transition behavior [73]. The quasi-1D structure of TMTCs makes them susceptible to phase transitions, such as CDW and SC [5,74–77].

**Table 2.** Comparison of crystal structure and bandgap data reported for TMTCs.

Materials	Crystal system	Crystal structure	Bandgap data (eV)		M_e_* (m_e0_) ^a^		M_h_* (m_e0_) ^a^	
			Bulk (Cal.)	Monolayer (Cal.)	*a* (Cal.)	*b* (Cal.)	*a* (Cal.)	*b* (Cal.)
TiS_3_	Monoclinic [62]	ZrSe_3_ type [62]	1.05 [63]	1.08 [63]	1.47 [37]	0.41 [37]	0.32 [37]	0.98 [37]
ZrS_3_	Monoclinic [62]	ZrSe_3_ type [62]	1.87 [63]	1.92 [63]	1.30 [37]	0.40 [37]	1.28 [37]	0.42 [37]
HfS_3_	Monoclinic [62]	ZrSe_3_ type [62]	1.90 [63]	1.94 [63]	–	–	–	–
NbS_3_	Monoclinic/Triclinic [3]	ZrSe_3_ type/NbSe_3_ type/TaSe_3_ type [59]	1.07 [63]/Metal [59]	1.18 [63]	–	–	–	–
TaS_3_	Monoclinic [59]	NbSe_3_ type [59]	Metal [61]	–	–	–	–	–
TiSe_3_	Monoclinic [59]	ZrSe_3_ type [59]	0.21 [37]/Metal [36]	0.57 [37]	0.19 [37]	4.29 [37]	3.57 [37]	0.85 [37]
ZrSe_3_	Monoclinic [59]	ZrSe_3_ type [59]	0.68 [63]	0.92 [63]	0.16 [37]	6.72 [37]	2.36 [37]	0.89 [37]
HfSe_3_	Monoclinic [59]	ZrSe_3_ type [59]	0.59 [63]	0.80 [63]	–	–	–	–
NbSe_3_	Triclinic [62]	NbSe_3_ type [62]	Metal [64,80]	–	–	–	–	–
TaSe_3_	Monoclinic [3]	TaSe_3_ type [3]	Metal [8]	–	–	–	–	–
TiTe_3_	Monoclinic [59]	ZrSe_3_ type [59]	Metal [36]	Metal [37]	–	–	–	–
ZrTe_3_	Monoclinic [59]	ZrSe_3_ type [59]	Metal [36]	Metal [63]	–	–	–	–
HfTe_3_	Monoclinic [59]	ZrSe_3_ type [59]	Metal [36]	Metal [63]	-	-	-	-

^a^M_e_* and M_h_* are the effective masses of electron and hole along *a*-axis and *b*-axis directions at the conduction band minimum and valence band maximum, respectively.

#### Charge density wave phases

The CDW is an exciting phenomenon in condensed matter physics, referring to the periodic modulation of electron charge density. The modulation of electron charge density opens a gap on the Fermi surface, decreasing or even disappearing electrical conductivity. The TaSe_3_ and NbSe_3_ family are typical CDW materials. The complexity of the Fermi surface can be reflected by the degree of inter-chain coupling and crystal symmetry. There are two stable CDW transitions at 144 K and 59 K in the NbSe_3_ crystal [78,79]. However, the transition only opens the energy gap on the partial Fermi surface, and the CDW transformed from NbSe_3_ still shows metallic behaviors [80,81]. For thick NbSe_3_ with a thickness of 20 to 170 nm, two stable CDW can be modulated by surface acoustic waves [82]. The investigation of the conductivity (*σ*) of NbS_3_ crystal and temperature (*T*) indicated that the high-density condensed charge shows low mobility under the temperature range of 450 to 475 K (the formation temperature of CDW) [83]. The band structure of TaS_3_ exhibits 1D metallic properties with Fermi level along the *Γ*-to-Y direction crossing 8 dispersive bands [61]. The CDW transformation can produce the metal–semiconductor transition in TaS_3_ under 210 K. In addition, the CDW current of these kinds of TMTCs is also affected by the photo-irradiation, magnetic field, and impurities [21,84–86].

The ZrTe_3_ is another well-known CDW material with a transition temperature of about 63 K [57,78,87]. As shown in Fig. 2B, ZrTe_3_ exhibits an anomaly in resistivity along the *a*-axis instead of *b*-axis, which is different from TaS_3_ and NbSe_3_ [87]. Hu et al. demonstrated that the phonon–electron coupling was essential for formatting CDWs in the quasi-1D ZrTe_3_ except for the instability of the Fermi surface [88]. In addition, the CDW transition can be observed at 93 K in a single crystal of HfTe_3_ [17]. As a semiconductor material, the metal–semiconductor transition around 220 to 250 K in TiS_3_ crystal can be detected, and the anomaly transmission phenomenon is related to the CDW transition [89–91].

#### Superconductivity

The CDW transition and SC are co-existent and competitive in quasi-1D TMTCs. The superconducting transition temperature of ZrTe_3_ is about 2 K under atmospheric pressure [92]. There is a highly anisotropic resistance transition in SC when the temperature is lower than the CDW transition temperature in ZrTe_3_ [57]. Resistance along the *a*-axis decreased at 4 K, while the resistance along the *b*-axis started to fall at 2 K. These differences are attributed to the SC caused by the locally bounded electron pairs, which is different from the traditional SC fluctuations. A similar phenomenon can also be observed in HfTe_3_ [17]. When the temperature is around 4 to 5 K, HfTe_3_ exhibits quasi-1D SC due to the superconducting pairings occurring along the *a*-axis. The SC transition is also in NbS_3_ around 2 K, which is influenced by the morphology [66]. The SC of the TMTCs can be improved by chemical element doping [33,78]. For example, the SC critical temperature of ZrTe_3_ can be increased via Se doped because of the suppressed long-range order of CDW (Fig. 2C) [78]. The novel topological SC has attracted considerable attention due to its unique physical properties, such as the capacity to carry Majorana Fermions. However, few topological SC materials have been reported [25]. Recently, the concomitant of SC and topological electronic structure has been observed in TaSe_3_, as displayed in Fig. 2D, which is considered a novel platform for investigating the topological SC [24,25,93–95]. The topological property-derived TaSe_3_ crystals can be attributed to the surface states near the terrace edge.

## Synthetic methods

The synthesis of TMTCs can be traced back to the 1960s [14]. Several preparation methods have been developed in order to control the morphology, scale, structure, and properties of TMTCs, such as CVT, CVD, ALD, and the solid-phase sulfurization method [3,28,38,44,96]. In the following section, we will summarize the recent progress of TMTCs synthesis along with their advantages and disadvantages. It should be noted that this review focuses on the comparisons of different synthesis strategies and novel growth methods for TMTCs materials. The detailed discussions on the growth parameters such as the reaction temperature, substrate, flow rate, and type of precursors can be found in previous reviews [3,34,35].

### Chemical vapor transport

**Fig. 3.**
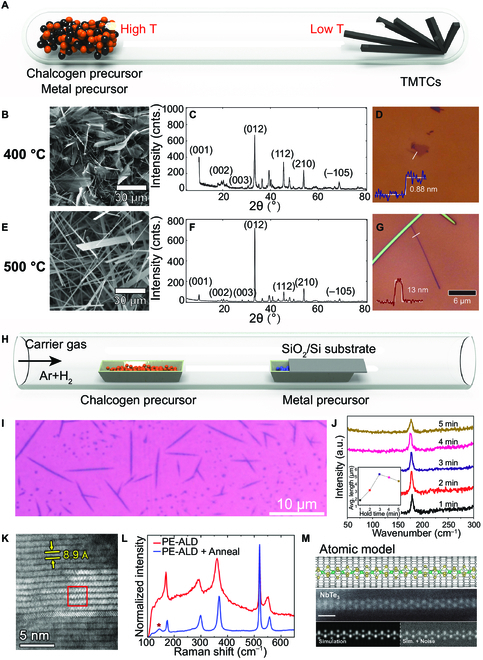
Fabrication methods of TMTCs. (A) Schematic representation of the chemical vapor transport (CVT) process inside a tube furnace. SEM images and XRD patterns of TiS_3_ grown at (B and E) 400 °C (nanosheets) and (C and F) 500 °C (nanoribbons) by the CVT method. (D and G) Optical images of TiS_3_ nanosheets and nanoribbons exfoliated from (B) and (E). Reproduced with permission [103]. Copyright 2015, Wiley-VCH. (H) Schematic representation of the chemical vapor deposition (CVD) process inside a tube furnace. (I) Optical image of TaSe_3_ nanowires prepared by the CVD method. (J) Raman spectra of TaSe_3_ nanowires were obtained by holding them at 400 °C at different times. The inset displays the resulting length distribution peaking at 3 min hold time. Reproduced with permission [113]. Copyright 2019, American Chemical Society. (K) High-angle annular dark-field scanning transmission electron microscopy (HAADF-STEM) image of the cross-section of postdeposition annealed TiS_3_. (L) The Raman spectra of plasma enhanced ALD (PE-ALD)-grown TiS_3_ before and after annealing at 400 °C. Reproduced with permission [115]. Copyright 2019, American Chemical Society. (M) Single-chain atomic structure, scanning transmission electron microscopy (STEM) imaging, and STEM simulation of NbTe_3_. Reproduced with permission [116]. Copyright 2020, American Chemical Society.

CVT is the primary preparation method for TMTC bulk crystals due to the advantages of large scale and high controllability. The transition metals and halogens are mixed with stoichiometric ratio and sealed in vacuum ampoules, as shown in Fig. 3A. Typically, I_2_, ICI_3_, S_2_Cl_2_, or TeCl_4_ was employed as transport agents in the transport process [3,38,68]. The ampoules were transferred into a certain temperature gradient during the growth process, while the reaction materials can be transported to the cooler side of the ampoules for crystal growth. As an ancient technique, CVT has been widely used for bulk TMTC material growth. Until now, TiS_3_, ZrS_3_, HfS_3_, ZrSe_3_, HfSe_3_, ZrTe_3_, and NbSe_3_ crystals have been synthesized via CVT. Different NbS_3_ structures can also be obtained by tuning the reaction temperature, time, pressure, and other factors [12,15,65,97–99]. Their reaction mechanisms are slightly different [3,12,34]. Due to the complex relationship between reaction factors and reaction processes, a thorough understanding of reaction mechanisms and details is necessary to accurately control crystal growth. Temperature is the most critical factor for the controllable growth of TMTCs. For example, Talib et al. [100] investigated the temperature-dependent morphology evolution of TiS_3_ with the stoichiometric ratio of Ti:S (1:3) under the temperature range of 400–650 °C. The results indicated that the morphology of TiS_3_ changed from nanosheets to nanoribbons (NRs) when the temperature increased from 400 to 550 °C (Fig. 3B to G). The TiS_3_ will be decomposed into TiS_2_ at a higher temperature. Similar results were also observed in the ZrS_3_ crystal [101].

Typically, the TMTCs synthesized via the CVT method are in bulk crystal. To further explore the potential in miniaturized, integrated, and flexible devices, bulk TMTCs have usually been exfoliated into few-layer or monolayer 2D or 1D crystals because of the low vdW force between layers. The theoretical interlayer cleavage energy of TiS_3_ crystal is 0.204 J m^−2^, which is lower than that of the graphene in graphite crystal (0.320 J m^−2^). Therefore, the bulk TiS_3_ crystals could be exfoliated into 2D layers or 1D chains [46,53].

Mechanical and liquid exfoliation are the primary methods for the bulk crystal. Mechanical exfoliation is an effective method and is widely used to exfoliate 2D layered materials. Since the first demonstration by Geim's group to prepare monolayer graphene in 2004, the mechanical exfoliation technique has been devoted to obtain few-layer materials, such as TMDCs and black phosphorus (BP). Mechanical exfoliation has recently been adopted to exfoliate bulk TMTCs, including TiS_3_, ZrS_3_, ZrSe_3_, ZrTe_3_, and NbSe_3_. The bulk crystals were exfoliated by tapes and transferred to specified substrates via dry or wet transfer method for further study [7,82,102–108]. Due to the uncontrollable morphology, thickness, and low yields, the mechanical exfoliated few-layer TMTCs have been limited to integration of electronic devices [28].

Beyond mechanical exfoliation, the liquid exfoliation method has largely improved exfoliation efficiency. Solvent direct exfoliation and ion intercalation exfoliation are two primary liquid exfoliation methods [28]. For the direct exfoliation method, few-layer or single-layer nanostructures are usually obtained by ultrasonic processing of the layered crystals in organic solvents. The matching between the solvents' surface tension and the layers' cleavage energy is vital to the exfoliation. Generally, organic solvents, including ethanol, isopropanol, *N*-methyl pyrrolidone (NMP), acetone, *N*,*N*-dimethylformamide, cyclohexylpyrrolidone, *N*-vinylpyrrolidone, and dimethyl sulfoxide, have been used in the liquid exfoliation process [28,45,89,109,110]. Baraghani et al. [89] had ultrasonically treated 100 mg of TiS_3_ whiskers in 50 ml of absolute ethanol under nitrogen protection to obtain needle-like TiS_3_ NRs. Similarly, Liu et al. added 30 mg of TiS_3_ bulk crystal into 30 ml of NMP and sonicated it under a thermostatic water bath at 15 °C for 6 h. After centrifugation and washing off the residual NMP, uniform TiS_3_ nanobelts with a thickness of about 30 nm were obtained [45]. The ion intercalation exfoliation method introduces excess ions into the interlayers of bulk TMTC materials, followed by ultrasonic treatment in a solvent to achieve exfoliation. In the 1970s, scientists reported that 3 lithium ions were inserted into the vdW interlayer spacing of TMTCs to form Li_3_MX_3_, while the MX_3_ chain structure remained [3]. The Li atoms can also be inserted into the region of the adjacent MX_3_ along the *a*-direction, leading to the exfoliated NRs in the process [4]. Both mechanical and liquid phase exfoliation can damage the morphology and surface structure of the material. More lossless stripping methods are needed to study the intrinsic properties of low-dimensional TMTCs, especially single- or few-chain ones.

### Chemical vapor deposition

The CVD method has been widely used in synthesizing various 2D layered materials, such as graphene, TMDCs, and other vdW heterojunctions. The schematic diagram is shown in Fig. 3H. The composition, doping, morphology, and thickness can be precisely controlled. Compared with CVT, the fast synthesis period and low vacuum environment requirement largely expand the universality of the CVD method and further meet the needs for industrialization [28,68,111]. However, there are few reports on the synthesis of 2D TMTCs via the CVD method, because it is still challenging to accurately introduce the stable amount of precursors and avoid the complex intermediate reaction. Yu et al. [112] proposed a modified CVD method to realize the rapid growth of ZrTe_3_ NRs. A confined space was formed by inserting wool in a small quartz tube of the CVD tube furnace. Utilizing ZrCl_4_ and tellurium powder as the zirconium and tellurium source, the geometric size of ZrTe_3_ NRs had been controlled by changing the growth temperature and time. Bartels’ group successfully prepared 1D vdW TaSe_3_ nanowires (NWs) via the CVD method using metal chloride adduct TaCl_5_[OEt_2_] for the match between the optimal growth temperature and the vapor pressure of tantalum and selenium precursors (Fig. 3I and J) [113]. Another example is from Sun et al. [114], in which TiS_3_ NRs and rectangular nanosheets were synthesized on mica substrates with thicknesses ranging from several nanometers to tens of nanometers. The aspect ratio of TiS_3_ NRs can be tailored by controlling the growth temperature. There were abundant S_2_^2−^ vacancies in the TiS_3_ NRs, resulting in high electrical conductivity and ultralow carrier activation barrier.

### Other synthetic methods

The ALD, solid-phase sulfurization method, and CNT packaging technology have also been adopted to synthesize TMTCs [44,115–117]. Basuvalingam et al. [115] utilized ALD to synthesize TiS_3_ at a lower growth temperature (Fig. 3K and L). The phase control was achieved by tuning the deposition temperature and copolymer composition. A rapid solid-phase sulfurization method has also been developed using S powder and Ti foil as precursor materials [44]. The advantage of this process is that it is fast and transfer-free. TiS_3_ NRs were synthesized by the sulfurization of the intermediate product TiS_2_. Stonemeyer et al. reported the synthesis of stable single-chain and few-chain NbTe_3_, VTe_3_, and TiTe_3_ by confining growth with multiwalled CNTs (Fig. 3M) [116,118]. The TMTC chain exhibited behaviors of few-chain quasi-1D structures, such as few-chain helical rotation and triangular antiprism rocking twist. However, CNTs have limited further research on their properties. Currently, these techniques have not been extensively studied and widely applied due to high cost and low efficiency. Exploring synthesis technologies for the efficient and controllable preparation of low-dimensional TMTCs is still challenging. The optimized exfoliation method, direct growth, and removal of CNTs should be studied intensively.

## Physical properties

### Electrical transport properties

**Fig. 4.**
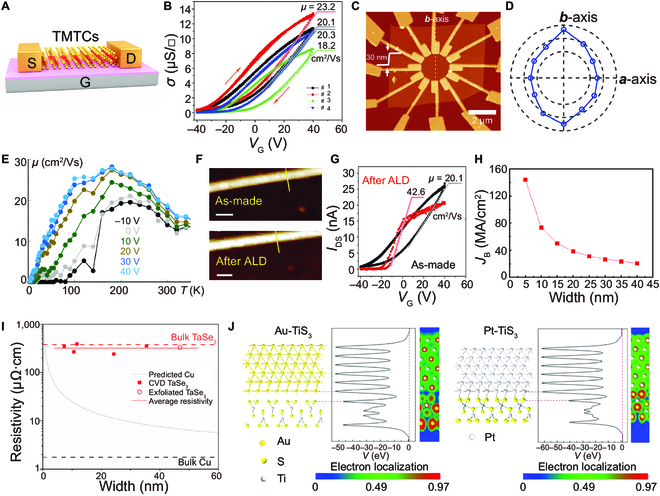
Electrical transport properties of TMTCs. (A) Schematic structures of field effect transistor with bottom gate structure. S, the source electrode; D, the drain electrode; G, the gate electrode. (B) Conductivity (σ)–gate voltage (*V*_G_) dependencies for different TiS_3_ FETs. Data were obtained for a drain-source voltage *V*_DS_ = 0.1 V. Reproduced with permission [122]. Copyright 2015, Royal Society of Chemistry. (C) and (D) are AFM images of the 30-nm TiS_3_ nanosheet FET device and the anisotropy of the mobility, respectively. *V*_G_ = −10 V; *V*_DS_ = 100 mV. The exterior and interior dashed lines in (D) mark the mobility of 80 cm^2^ V^−1^ s^−1^ and 40 cm^2^ V^−1^ s^−1^, respectively. Reproduced with permission [103]. Copyright 2015, Wiley-VCH. (E) The mobility of TiS_3_ nanowire FET as a function of temperature. Data were obtained for a drain voltage *V*_d_ = 100 mV. Reproduced with permission [90]. Copyright 2018, American Chemical Society. (F) AFM image of TiS_3_ nanoribbon channel and after atomic layer deposition (ALD) of 30 nm of Al_2_O_3_. Scale bars are 100 nm. (G) The drain-source current (*I*_DS_)–*V*_G_ dependencies for the device before and after ALD of Al_2_O_3_. *V*_DS_ = 0.1 V. Reproduced with permission [122]. Copyright 2015, Royal Society of Chemistry. (H) The functional relationship between the breakdown current density and the width of ZrTe_3_ nanoribbons was obtained by finite element simulation. Reproduced with permission [127]. Copyright 2021, American Chemical Society. (I) Resistivity of TaSe_3_ nanowire bundles as a function of bundle width. Reproduced with permission [113]. Copyright 2019, American Chemical Society. (J) Side view of the atomic structures (left) and effective potentials (EPs) (right), as well as electron localization function (ELF) (rightmost) along the perpendicular to the interface with ML TiS_3_ on Au and Pt, respectively. The Fermi level is at zero energy. Reproduced with permission [131]. Copyright 2019, American Chemical Society.

The theoretical calculations of quasi-1D TMTCs indicate that the monolayers of MS_3_ and MSe_3_ (M = Ti, Zr, Hf, Nb) exhibit semiconducting properties, while MTe_3_ exhibits metallic behaviors [63]. Compared with TMDCs, the 1D chain structure of TMTCs is effective in improving or suppressing the edge scattering of carriers, which is crucial for the preparation of high-performance nanoelectronic devices [41]. The carrier mobility of bulk TiS_3_ is calculated to be 30 cm^2^ V^−1^ s^−1^ at room temperature (RT) and around 100 cm^2^ V^−1^ s^−1^ with a temperature lower than 100 K [119]. Dai and Zeng [39] predicted that the carrier mobility of monolayer TiS_3_ was highly anisotropic, where the electrons were the main carrier. Theoretical calculations of TiS_3_ monolayers show that the coupling of the electronic bands is strongest along the chain direction, while the coupling of the hole bands is the strongest along the vertical chain direction. Electrons are more likely to propagate along the 1D chain and exhibit high mobility. The electron mobility along the *b*-axis of the 1D chain direction can reach 10^4^ cm^2^ V^−1^ s^−1^, which is one magnitude higher than monolayer MoS_2_ (1,000 cm^2^ V^−1^ s^−1^). The experimental results demonstrate that the anisotropy ratio of the conductivity of few-layer TiS_3_ nanosheets is 2.1 at RT. The value increased to 4.4 at 25 K, making TiS_3_ an ideal material for high-performance field-effect transistors (FETs) [7,120,121]. We noted that the carrier mobility of TiS_3_ measured using FETs is much lower than that of the theoretical calculation, as shown in Figs. 4A and 3B [7,90,122]. Island et al. [103] investigated the in-plane transport performance of TiS_3_ nanosheets with a thickness of 30 nm. The highest carrier mobility was 73 cm^2^ V^−1^ s^−1^ along the *b*-axis, which is two orders of magnitude lower than the theoretically calculated value (Fig. 4C and D). They ascribed the low performance of TiS_3_ FET to the large number of sulfur vacancies in the nanosheets, which could be improved effectively by reducing them [103]. In addition to the sulfur vacancies, some other factors greatly influenced the device mobility, such as the thickness and surface roughness of TMTC materials, structural defects in bulk crystals, temperature, and geometric parameters (Fig. 4E) [4,90,121,123,124]. For example, monolayer NRs have much lower carrier mobility than nanosheets due to the width-dependent variation [121].

The reduction of external scattering has been proven effective in improving the carrier mobility of 2D graphene and TMDCs [68,122,125,126]. To verify the applicability of this method to TMTCs, Lipatov et al. deposited 30 nm of Al_2_O_3_ on mechanically exfoliated single TiS_3_ NRs to prepare FET (Fig. 4F). The carrier mobility of the device increased from 20.1 cm^2^ V^−1^ s^−1^ to 42.6 cm^2^ V^−1^ s^−1^, while the on/off ratio increased from 300 to 7,100 (Fig. 4G) [122]. High current density caused electrical breakdown for miniaturized devices. Therefore, the breakdown current density needs to be considered to evaluate the applications of TMTC materials. It is predicted that the breakdown current density of ZrTe_3_ NRs can be 144 MA cm^−2^, which has the potential to be a novel interconnect material for the next generation of micro-integrated circuits (Fig. 4H) [127]. TaSe_3_/h-BN NWs heterostructures with a high aspect ratio (widths of 20–70 nm) and Nb_1−*x*_Ta*_x_*S_3_ nanofibers also have high breakdown current density (10 and 30 MA cm^−2^, respectively), which is an order of magnitude higher than copper (Cu) (Fig. 4I) [8,113,128]. The excellent breakdown current density can be attributed to the unique crystal structure of the quasi-1D vdW material [9,129]. The breakdown current density of TiS_3_ NRs also reaches 1.7 MA cm^−2^, which is higher than most reported nanomaterials [130]. The electrical breakdown of TiS_3_ NRs was caused by crystal defects formed by material oxidation and sulfur atom desorption. The TMTCs with high breakdown current density are promising for miniaturized nanoelectronic devices.

The interfacial properties of TMTCs and metal contacts have also been investigated, which are essential for advanced electronic devices [131–133]. For example, Gilbert et al. [132] evaluated the electronic properties of Au and Pt metal contacts on (001) planes of TiS_3_ via x-ray photoelectron spectroscopy (XPS). The results indicated that an ohmic contact, instead of the Schottky barrier, is formed at the interface of Au and the TiS_3_ (001) plane. It was believed element S plays a vital role in this process. The DFT calculations demonstrated that there is no tunneling barrier between the monolayer TiS_3_ and 6 metals (Ag, Au, Pt, Pd, Ir, and Ni), which indicated that high carrier injection efficiency was achieved from metal to semiconductor [131]. However, the band structure of the monolayer TiS_3_ was affected by the metal of Pd, Pt, Ir, and Ni, which will form covalent bonds at the interface to metalize the semiconductor (Fig. 4J).

### Optical properties

The optical properties of TMTCs exhibit high anisotropy due to the quasi-1D structure. The interaction between TMTCs and light, and corresponding spectral characteristics (i.e., absorption, emission, scattering, luminescence, and refraction) will be discussed as follows [7,46,71,134–145].

Linear dichroism refers to the distinctive absorption of polarized light perpendicular to or parallel to an orientation, which is an essential index for evaluating polarization-dependent optical properties [11,135,146,147]. The dichroic ratio for the (001) plane of TiS_3_ and the (001) plane of ZrS_3_ can reach 4 and 2.55 in the photocurrent measurement, respectively [136,146 ,147]. The angle-resolved photoemission spectroscopy (ARPES) indicates that the strong polarization sensitivity of TiS_3_ and ZrS_3_ is caused by the different in-plane symmetries of their electronic energy bands. The optimal excitation energy of TiS_3_ photocurrent dichroism is 1.0 to 2.0 eV. The excitons with larger binding energy are the primary photoexcitation in TiS_3_ NRs [134]. In the ultrahigh-speed injection state, the exciton can be formed in a subpicosecond time scale with a mobility of 50 cm^2^ V^−1^ s^−1^.

**Fig. 5.**
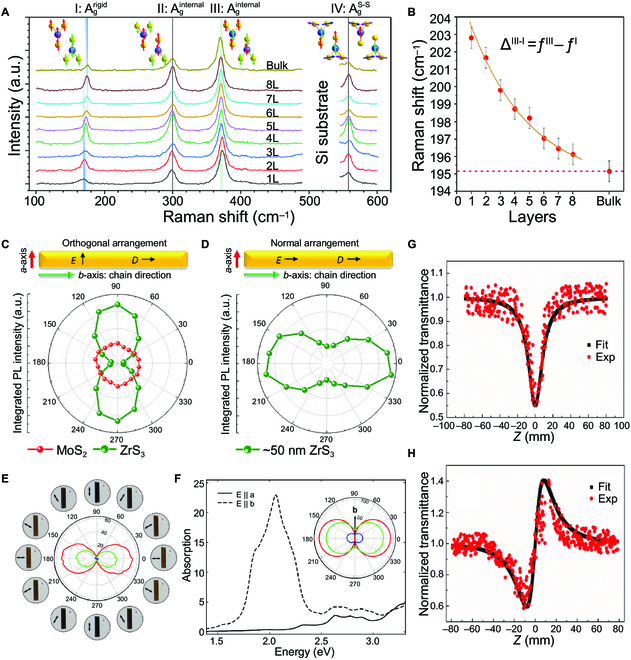
Optical properties of TMTCs. (A) Raman spectra of TiS_3_ nanosheets with different thicknesses. (B) Raman-active modes in (A) as a function of the number of layers. Reproduced with permission [46]. Copyright 2018, American Chemical Society. (C) and (D) are the polar plot of the integrated peak area with azimuthal ZrS_3_ flake angle in orthogonal arrangement and in normal arrangement, respectively. *E*, the electric field direction of the polarized excitation laser. *D*, the polarization direction of the detector. Reproduced with permission [137]. Copyright 2016, Royal Society of Chemistry. (E) The transmittance of TiS_3_ nanosheets in red, green, and blue channels as a function of the excitation polarization angle. (F) Computed absorption spectra of TiS_3_ when the field is aligned parallel to the *a*-axis (solid line) and *b*-axis (dashed line). The inset shows the transmittance for energies blue (2.72 eV), green (2.4 eV), and red (1.9 eV) excitations in the *a*–*b* plane. Reproduced with permission [7]. Copyright 2016, Nature Publishing Group. (G) and (H) are nonlinear absorption and nonlinear refractive performance of ZrS_3_ nanobelts with the input energy of 34.25 μJ, respectively. Reproduced with permission [140]. Copyright 2016, Royal Society of Chemistry.

Similar to TMDCs, Raman spectroscopy can be used to distinguish the layer number of thin TMTC crystals [46,142]. For example, the TiS_3_ has 4 *A_g_* Raman activity modes in the range of 100 to 600 cm^−1^, including A_g_^rigid^ (175 cm^−1^), A_g_^internal^ (300 cm^−1^), A_g_^internal^ (370 cm^−1^), and A_g_^S-S^ (560 cm^−1^), as shown in Fig. 5A [46]. With the decrease in layers, the intensity of A_g_^rigid^ decreases, while the intensity of the A_g_^internal^ increases. On the contrary, the intensity of A_g_^internal^ and A_g_^S-S^ remain constant despite the changed layer number (Fig. 5B). Therefore, the TiS_3_ number can be identified using the deviations of peak positions between the A_g_^internal^ and A_g_^rigid^. Similar behavior can also be observed in ZrS_3_ and ZrSe_3_ [142].

TMTCs have ultrahigh luminescence, which is 5 times higher than BPs, and 10 times higher than ReS_2_ at the same thickness [137]. The strong photoluminescence (PL) peaks of high-aspect-ratio HfS_3_ NRs were observed at 485, 540, and 600 nm under an excitation wavelength of 400 nm [139]. The angle resolved PL spectroscopy of ZrS_3_ flakes indicates that light is efficiently absorbed, and the maximum PL intensity appears when the electron field is polarized along the *b*-axis chain direction [137]. When the electric field is perpendicular to the *b*-axis, the absorption decreases because the wavelength of the excitation light is much longer than the chain width (Fig. 5C and D). The dichroic ratio of PL intensity reached 10.8, similar to 1D materials. The anisotropy of polarization is smaller than 1D materials. The strongly isotropic PL emission of thermally oxidized ZrS_3_ NRs has also been investigated, comparable to monolayer direct bandgap semiconductor MoS_2_ [141]. Uniform ZrO_2_ nanocrystals were formed on the surface of ZrS_3_ after thermal oxidation treatment, providing additional degrees of freedom for electro-optical modulation of TMTCs.

The TiS_3_ nanosheets had large birefringence, which is larger than the well-known strong birefringence materials, such as TiO_2_ and calcite [143]. The exciton effect is believed to play an important role in the large birefringence. The birefringence and linear dichroism of ZrS_3_ were investigated by polarization-resolved optical microscopy and azimuth-dependent reflectometry microscopy [138]. It was found that the refractive indices and extinction coefficients have different peak values and change trends along the *a*-axis and *b*-axis of ZrS_3_.

The light transmittance measurements show that TiS_3_ has a transmittance of 30, which is much larger than other 2D anisotropic materials such as MoS_2_ (1) and BP (1.4) (Fig. 5E) [7]. It is because the TiS_3_ NRs have a quasi-1D chain-like structure. When the excitation light is polarized along the *b*-axis chain direction, there will be an absorption similar to wire grid polarizers, resulting in a minimized transmittance (Fig. 5F).

Nonlinear optical properties of ZrS_3_ NRs have also been reported [140]. The nonlinear refractive index (*γ*) and nonlinear absorption coefficient (*β*) of ZrS_3_ NRs in ethanol are *γ* = 5.86 × 10^−17^ and *β* = 4.42 × 10^−10^ mW^−1^, respectively, under the laser-pulsed energy of 34.25 μJ (Fig. 5G and H). The ZrS_3_ NRs composited with graphene and reduced graphene oxide exhibit a more robust nonlinear absorption response, which makes ZrS_3_ NRs promising for novel optical power confinement applications.

### Thermoelectric properties

The thermoelectric conversion efficiency of a material is determined by the quality factor value of *ZT* (*ZT* = *S*^2^*σT*/*κ*), where *S*, *σ*, *T*, and *κ* are Seebeck coefficient, electrical conductivity, absolute temperature, and thermal conductivity, respectively [28,49]. These parameters are controlled by phonon scattering and electronic structure. High power factor (*S*^2^*σ*) and low κ are favorable for ideal thermoelectric materials.

**Fig. 6.**
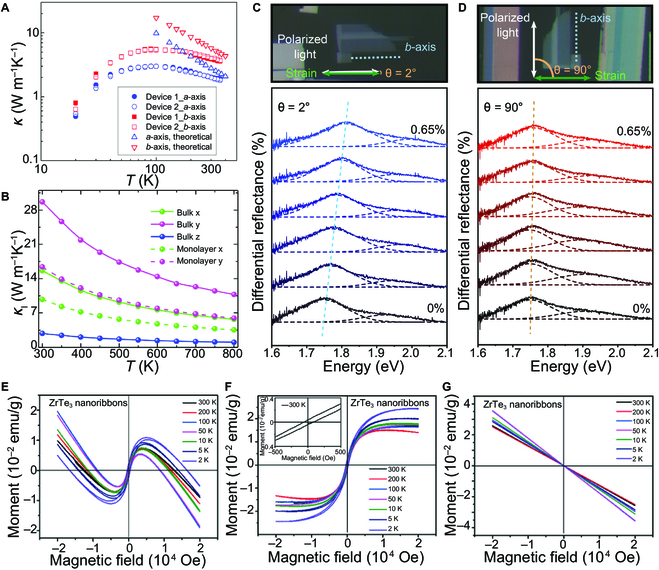
Thermoelectric properties, mechanically induced properties, and magnetic properties of TMTCs. (A) Temperature dependence of thermal conductivity (κ) along the *a*- and *b*-axis of TiS_3_. Reproduced with permission [150]. Copyright 2020, American Chemical Society. (B) Temperature dependence of phonon thermal conductivity for bulk and monolayer ZrS_3_. Reproduced with permission [49]. Copyright 2020, American Chemical Society. (C) and (D) are the angle-resolved micro-reflectance spectra obtained when the uniaxial strain direction is parallel to the *b*- and *a*-axis of the ZrSe_3_ flakes, respectively. Reproduced with permission [105]. Copyright 2021, Wiley-VCH. (E) M–H curves of the ZrTe_3_ nanoribbons at different temperatures in the magnetic field range of −20,000 to 20,000 Oe. (F) and (G) show the deconvoluted curves of the ferromagnetic and diamagnetic characteristics corresponding to (E), respectively. The inset of (F) shows an enlarged view of the M–H curve at 300 K, where a distinct hysteresis loop with a coercivity of ∼87 Oe can be observed. Reproduced with permission [112]. Copyright 2019, Royal Society of Chemistry.

There are many theoretical and experimental reports on the thermoelectric property of monolayer TiS_3_ and ZrS_3_ [31,49,120,148–152]. The extreme anisotropy exists in the in-plane thermal conductivity of TiS_3_ material. The thermal conductivity along the *b*-axis is twice higher than the *a*-axis, higher than the reported other layered materials, as shown in Fig. 6A [150]. The large dispersion of optical phonons in the chain direction is the principal cause for the high anisotropic thermal conductivity of TiS_3_. The thermal conductivity of monolayer TiS_3_ is much lower than TMDCs, which results in a high *ZT* value of 3.1 under moderate carrier concentration at RT [120]. Both high Seebeck coefficient and electrical conductivity can be obtained in monolayer ZrSe_3_ due to the grooved band near the conduction band minimum [51]. The n-type *ZT* value of monolayer ZrSe_3_ with moderate carrier concentration is 2.4 at 800 K. The selenium atoms on the surface of ZrSe_3_ play a major role in the heat transport process. The *ZT* value of monolayer ZrS_3_ along the *b*-axis direction is predicted to be 2.44 at 800 K, ascribed to the excellent thermoelectric performance similar to single-layer ZrSe_3_ [49]. There have been several reports on the theoretical calculation of the thermoelectric properties of TMTC materials. For example, Wang et al. [48] analyzed the thermoelectric properties of bilayer ZrS_3_ and Janus ZrS_2_Se using the first-principle calculation method. The results indicated that ZrS_3_ had a higher power factor and lower lattice thermal conductivity. The thermoelectric properties were improved by partially replacing S atoms with Se atoms. Under 300 K, the optimal *ZT* values of p-type and n-type doped bilayers Janus ZrS_2_Se are 2.21 and 1.43, respectively, which are higher than bilayer ZrS_3_.

The thermoelectric properties of TMTCs are correlative with the thickness. The phonon confinement effect will dominate when the thickness of the TMTCs is smaller than the phonon confinement size. Due to the increased phonon scattering, the in-plane thermal conductivity is negatively correlated with thickness [31,153]. In other words, the phonon thermal conductivity can be effectively decreased by reducing the dimensionality of the crystal structure. It is confirmed that the thermoelectric performance of monolayer ZrS_3_ is superior to its bulk crystal, as shown in Fig. 6B [49]. A 25-fold enhancement of *κ* was observed in NbSe_3_ NWs when the diameter was diminished from 26 to 6.8 nm, providing strong experimental evidence for 1D phonon transport [153].

Metallic TaSe_3_ and ZrTe_3_ with high breakdown current density have been used as connecting channels in nanoscaled electronics. The Joule heating effect of NWs will cause the performance degradation of devices. Thus, their thermal transport properties need to be considered. A theoretical study indicates that the thermal conductivity of TaSe_3_ and ZrTe_3_ from chain direction is higher than cross-chain and cross-plane directions [154]. The phonon lifetime and mean free path of TaSe_3_ are shorter than ZrTe_3_ in the low-frequency range. The NbSe_3_ and TaS_3_ materials exhibit metallicity at RT, while the Peierls transition occurs at low temperature, complicating the electrons' thermal conduction in these two NWs [22,47,155].

### Mechanically induced properties

Strain engineering has been proven effective in tuning materials' intrinsic properties. The changes in the lattice structure of TiS_3_ and ZrS_3_ have been observed under a larger pressure [53,54,56,156]. Specifically, distortion occurred in multilayer ZrS_3_ crystal when the pressure reached 2.5 GPa [53]. Further, when the pressure was increased to 10.8 GPa, the S–S bonds were rearranged along the *a*-axis, resulting in a phase transition in the ZrS_3_ crystal. During this process, the original S–S bonds connected to the Zr atoms are broken and reconnected to the S atoms on the adjacent chain, leading to a new S–S bond. The results were confirmed by high-pressure Raman spectroscopy [53]. A similar phenomenon has been observed in TiS_3_ crystals, in which the TiS_3_ crystals changed from monoclinic to cubic phases under large pressure [156]. The cubic phase exhibits superconducting potential with an estimated transition temperature of 9.3 K at 80 GPa.

The effect of stress on atomic displacements in crystals can also be observed in the optoelectronic properties of TMTC materials. The DFT calculations indicate that the electronic structure of the MX_3_ monolayer can be affected by tensile strain [63,157]. Specifically, monolayers of HfS_3_ and ZrS_3_ could be transformed from an indirect bandgap to a direct bandgap under a tensile strain of *ε* = 2%. Further, when *ε* = 6% and *ε* = 4% strains are applied, the monolayer HfTe_3_ and ZrTe_3_ will transform from intrinsic metallic into indirect bandgap semiconductors. On the contrary, the monolayer TiS_3_ and NbS_3_ could keep the bandgap feature under monoaxial and biaxial tensile strains ε ranging from 0% to 8%. The calculation results show that the bandgap transition occurred more easily in monolayer MX_3_ than MX_2_ under external strain. Experimental studies have also observed an increased bandgap (up to 9%) when tensile stress is applied to TiS_3_ along the *b*-axis [40]. The monolayer and bilayer TiS_3_ change from direct to indirect bandgap semiconductor when the compressive strain is generated. Silva-Guillén et al. [70] believed that the change of the energy band in monolayer TiS_3_ under strain could be attributed to the interaction between Ti 3d_xy_ and S 3p_x_ orbitals. Thus, the strain can be used to manipulate the anisotropy of TiS_3_ materials. The strain effect on the bandgap of monolayer TiS_3_ has also been used to tune its optical properties to expand the light absorption range [157]. Anisotropic experimental results have been observed when monoaxial strain experiments are performed on ZrSe_3_ using a 3-point bending test apparatus [105]. Specifically, when the strain was applied along the *b*-axis, an apparent blue shift of the exciton peak (≈ 60 to 95 meV %^−1^) was observed (Fig. 6C). In contrast, the phenomenon did not appear along the *a*-axis (≈ 0 to 15 meV %^−1^) (Fig. 6D). Lin and colleagues utilized ultrahigh-resolution ARPES to study the electronic structure evolution of TaSe_3_ under tensile strain [95,158]. They observed metal–insulator transition on the stressed TaSe_3_.

Both monoaxial and biaxial tensile strains have been theoretically proven to improve the intrinsic mobility of the TMTCs [157]. The results showed that an order of magnitude had increased both the electron mobility and the hole mobility of monolayer TiS_3_. TiS_3_ nanosheets exhibited anisotropic piezoresistive effects [159]. The SC of ZrTe_3_ was also affected by pressure [55,57]. The superconducting transition temperature of ZrTe_3_ increased from 4 K to 7.1 K when the pressure increased to 28 GPa. Localized electronic states near the Fermi level in wrinkled HfTe_3_ film are anticipated to enhance the SC transition temperature [160]. The CDW transport performance of MX_3_ can also be modulated by mechanical force [19,52,161]. The CDW will periodically stop when the piezoelectric actuator's vibration frequency coincides with the resonance of the TaS_3_ whiskers [52].

### Magnetic properties

It is believed that TMTCs exhibit diamagnetic properties [112,162]. The weak magnetism has been found in ZrTe_3_ crystal, and the corresponding Curie temperature is higher than 300 K (Fig. 6E to G) [112]. There is an interaction between ferromagnetism and diamagnetism in the ZrTe_3_ crystal. The hysteresis loop indicated that ferromagnetism plays a dominant role under the magnetic field of −5,000 Oe to 5,000 Oe. The diamagnetism will be dominant beyond the magnetic field range. This RT ferromagnetism can be ascribed to the structural defects and size-reduced edge states of ZrTe_3_. The introduction of structural defects, such as vacancies and grain boundaries, has been reported to be an effective method of generating the local magnetic moments [28,163].

The DFT studies indicate that the magnetic properties are highly dependent on the crystallographic orientation of TMTC materials. The first-principles study on TiS_3_ NRs has shown that the *a*-TiS_3_ NRs (grown along the *a*-axis) exhibited ferromagnetic metallicity properties, while the *b*-TiS_3_ NRs (grown along the *b*-axis) were the nonmagnetic direct bandgap semiconductor [121]. The net magnetic moment of the *a*-TiS_3_ unit cell changed between 0.2 μB and 0.8 μB under different widths of NRs. The spin-polarized state originates mainly from the unpaired electrons of the band-edge atoms. The discovery of TiS_3_ ferromagnetism is supposed to be helpful for the fabrication of spintronic devices. The related results provide new ideas for further study of the magnetic properties of other TMTCs.

## Applications of TMTCs

### Photodetectors

**Fig. 7.**
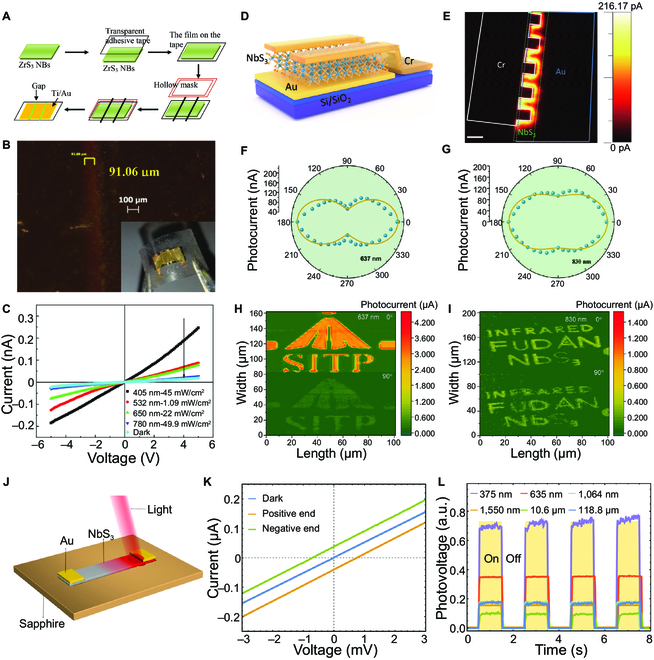
Photodetectors of TMTCs. (A) Schematic diagram of the fabrication of the ZrS_3_ nanobelt flexible photodetector. (B) Optical micrograph of the 2-channel ZrS_3_-nanobelt photodetector with a width of 91.06 μm between the electrodes. Inset is an optical image of the photodetector on a polypropylene film substrate. (C) *I*–*V* curves of photodetectors under dark conditions and different wavelength illumination conditions. Reproduced with permission [169]. Copyright 2014, Wiley-VCH. (D) Schematic of NbS_3_ Schottky photodetector. (E) Spatially resolved photocurrent mapping of the NbS_3_ Schottky photodetector under an 830-nm illumination at 0 V bias. The green dotted lines show the 2D NbS_3_. The blue and white lines are Au and Cr electrodes, respectively. The scale bar is 5 μm. Polar plot of the normalized polarization-dependent photocurrent under 637-nm (F) and 830-nm (G) illumination at 0 V bias. Polarization imaging of NbS_3_ photodetector via reflective mode at 0° and vertical 90° under 637-nm (H) and 830-nm (I) illumination at 0 V bias. Reproduced with permission [172]. Copyright 2020, Wiley-VCH. (J) Schematic of NbS_3_-based photothermoelectric (PTE) detector. (K) *I*–*V* characteristics of the device in (J). (L) On–off curves of the photovoltage of the NbS_3_-based PTE detector at room temperature, normalized with the same incident power. Reproduced with permission [10]. Copyright 2020, American Chemical Society.

The TMTCs are promising candidates for optoelectronic devices due to their novel physical and chemical properties such as tunable bandgap. There have been several reports on photodetectors based on TMTCs, such as TiS_3_, ZrS_3_, ZrSe_3_, and NbS_3_ (Table 3) [42,102 ,164 –167]. Few-layer TiS_3_ NRs have distinct response signals to wavelengths in the visible spectrum, enabling high and fast (4 ms) photoresponse up to 2,910 A W^−1^ [102]. The TiS_3_ NRs can be horizontally aligned on the interdigital electrodes by the dielectrophoresis method [164]. The TiS_3_ NR-based photodetector exhibited a large detection range with extension to the near-infrared region. The maximum responsivity, quantum efficiency, and detection rate of TiS_3_ NR-based photodetector were 5.22 × 10^2^ A W^−1^, 6.08 × 10^2^, and 1.69 × 10^9^ Jones, respectively. The comparative experiments suggested that the photoresponse of horizontally aligned NRs was higher than the randomly oriented NRs. The ZrS_3_ NR-based phototransistors fabricated on SiO_2_/Si wafers exhibited remarkable photoresponse from ultraviolet to near-infrared light [165]. The optical power of ZrS_3_ NRs was 5.57 mW cm^−2^ at a bias voltage of 1 V under 405 nm. The photoswitch current ratio can reach 210, while the photoresponse time was less than 0.4 s. The photodetector based on single HfS_3_ NR exhibited selectivity photoresponse to 405 nm with a low dark current of 0.04 pA and a large photoswitching current ratio of 337.5 [168]. The aforementioned ZrS_3_ and HfS_3_ are p-type semiconductors, and holes are the prominent carriers. For p-type semiconductors, the adsorbed oxygen captures photogenerated electrons under light exposure and increases the conductivity and photocurrent of the TMTCs. In contrast, the adsorbed oxygen decreases under vacuum or nitrogen protection, leading to a decreased photocurrent. The result is opposite to the n-type TiS_3_ photodetector. The flexible UV-vis photodetectors based on ZrS_3_ NR, HfS_3_ NR, and TiS_3_ fake films were fabricated on transparent polypropylene films and printed paper substrates (Fig. 7A to C) [139,169 ,170]. Though the performance of the flexible photodetectors is not comparable to that on SiO_2_/Si substrates, they can withstand mechanical forces. In addition, spectral selectivity and wide spectral detection range can be maintained, as well as excellent environmental stability, which is expected to expand the applications.

**Table 3.** Performance of TMTC-based photodetectors.

Materials	Response range	Responsivity	Response time	Bias	Ref.
TiS_3_ NR	1,130 nm	2,910 A W^−1^	4 ms	–	[102]
Horizontally aligned TiS_3_ NRs	1,064 nm	522 A W^−1^	1.53 s	12 V	[164]
ZrS_3_	520 nm	230 mA W^−1^	–	3 V	[147]
ZrS_3_ NR	405 nm	–	0.42 s	3 V	[169]
	405 nm	0.36 A W^−1^	<0.4 s	1 V	[165]
ZrS_3_ single crystal	Visible light	0.120 mA W^−1^	1.8 s	1 V	[171]
ZrSe_3_ NR	650 nm	0.53 A W^−1^	<0.4 s	5 V	[166]
HfS_3_ NRs	405 nm	0.11 A W^−1^	<0.4 s	- 5 V	[168]
	405 nm	–	0.2 s	5 V	[139]
HfSe_3_ NR	532 nm	0.012 A W^−1^	<0.4 s	5 V	[166]
NbS_3_	UV−THz	1.06−6.9 V W^−1^	∼7 ms	0	[10]
NbS_3_ Schottky junction	830 nm	0.025 A W^−1^	11.6 μs	−1 V	[172]
TiS_3_/Si heterojunction	405 to 1,050 nm	0.33 to 34.8 mA W^−1^	<20 ms	−0.5/0 V	[173]
PbS/TiS_3_ heterostructure	300 to 1,300 nm	0.36 A W^−1^	–	0 V	[174]

The low symmetric structure and high anisotropy performance of TMTCs make them promising in polarization photodetection [171]. The indirect narrow-bandgap semiconductor NbS_3_ has a bandgap of 0.26 eV and 0.42 eV for bulk and monolayer, respectively. 2D NbS_3_ Schottky detectors were fabricated with asymmetric electrodes of Cr and Au, as displayed in Fig. 7D and E [172]. High linear dichroic ratios (3.95 and 1.84) and high-quality polarization images were obtained at 637 and 830 nm, respectively (Fig. 7F and G). In addition, the detection rate exceeds 10^7^ Jones at a wavelength of 3 μm under RT. Fast photoresponse (11.6 μs) and lower noise current (4.6 × 10^−25^ A^2^ Hz^-1^) had also been achieved. The photovoltaic effect is the key feature of the photodetector in the ranges from visible to near-infrared, and the built-in electric field separates the photogenerated carriers in the space-charge region, while the PL thermal effect is dominated in the mid-infrared to the long-wave infrared band. The temperature of the device increases with the illumination, which leads to the enhancement of the hot carriers and the increase of the electrical conductivity. The measurements show that the dichromatic ratios of TiS_3_ and ZrS_3_ reach 4 and 2.55, respectively, exhibiting high sensitivity to polarized light [136,147]. Wang et al. [147] found that ZrS_3_ NRs had maximum light absorption along the *b*-axis chain direction, and the dichroic response was affected by the excitation wavelength. Under the light irradiation of 450 nm, the dichroic ratio was 1.73, while the ratio reduced to 1.14 when the wavelength changed to 532 nm. The tunable dichroic response was attributed to the higher exciton absorption peak of ZrS_3_ than 450 nm, which could effectively detect exciton absorption. Meanwhile, the polarization sensitivity of ZrS_3_ NRs is dependent on the layer number, the intrinsic band structure, and the optical transitions.

Self-powered devices without an external power supply have been of great interest in recent years due to their low power consumption, light weight, and small size. Generally, the p-n junction and Schottky junction have been used to realize self-powered devices. The built-in electric field is generated inside the device due to the separation of carriers. Self-powered photodetectors based on p-n junctions have the advantages of fast response, large linear regions, and low noise. The TiS_3_/Si p-n junction monolithic device can operate in the photovoltaic mode without external bias, or photoconductive mode with a positive or negative bias [173]. Yao et al. constructed a self-power photodetector in a 1D solar cell capacitance simulator (SCAPS-1D) software [174]. The fluorine-doped tin oxide FTO, PbS, and narrow-bandgap TiS_3_ were indicated as the transparent conductive layer, donor, and acceptor, respectively. The offset value of the conduction band between PbS and TiS_3_ is about 0.6 eV, which helps the photogenerated electrons move into the Ag electrode. The photogenerated holes were transmitted to the FTO layer due to the shift of the valence band. The simulation results indicated that the PbS/TiS_3_ photodetector with the optimal parameters exhibited a responsivity and detection limit of 0.36 A W^−1^ and 3.9 × 10^13^ Jones, respectively. The photodetectors based on the photothermoelectric (PTE) effect can also realize self-driven operation under zero bias voltage. The effect derives from the asymmetry of the electrode material or the temperature distribution along the channel direction. Utilizing the thermally localized enhanced PTE effect, Wu et al. [10] fabricated a quasi-1D flexible NbS_3_ photodetector with broadband detection from ultraviolet (375 nm) to terahertz (118.8 μm) band (Fig. 7J to L). When the light was illuminated at one end of the device, the generated heat produced a temperature gradient at both ends. Since the thermal decay length of the NbS_3_ material is short, the heat generated by the illumination was confined to a small area, which resulted in a large temperature difference between the two ends of the device. Under various wavelengths, the optical radiation rate of the device was higher than 1 V W^−1^ and the response time was less than 10 ms. The flexible NbS_3_ photodetector on PET substrate also demonstrated great stability, showing no obvious degradation of photoresponse performance after 150 bending cycles.

### Energy storage

**Fig. 8.**
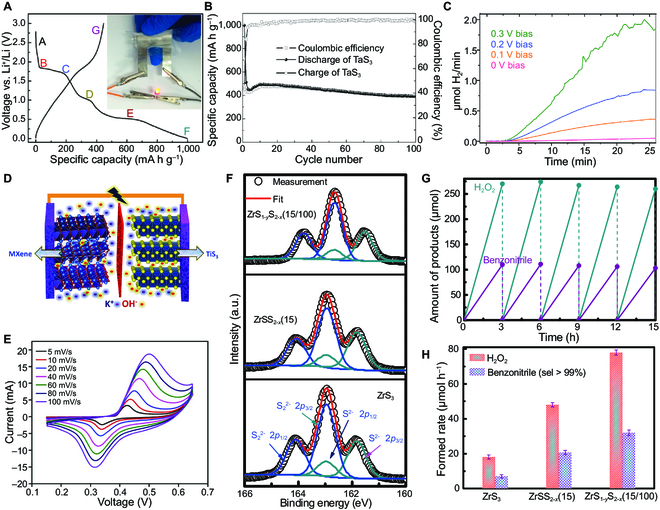
Energy storage and catalysis of TMTCs. (A) Discharge/charge profiles of a lithium-ion battery based on TaS_3_ nanowires for the first cycle. Inset is the optical image of a red glowing LED powered by the TaS_3_ nanowires flexible battery. (B) Capacity and Coulomb efficiency of TaS_3_ nanowire electrode at a cycling rate of 0.1 C as a function of the number of cycles. Reproduced with permission [176]. Copyright 2015, American Chemical Society. (C) Hydrogen evolution properties of TiS_3_ NRs under different bias potentials. Reproduced with permission [181]. Copyright 2015, Royal Society of Chemistry. (D) Schematic diagram of the charge storage mechanism of the asymmetric TiS_3_ device. (E) CV curves at various scan rates of TiS_3_. Reproduced with permission [183]. Copyright 2022, AIP Publishing. (F) S 2p XPS spectra of the ZrS_3_, ZrSS_2-*x*_(15) and ZrS_1-*y*_S_2-*x*_(15/100) NRs. (G) Repeatability of the catalytic production of H_2_O_2_ and benzonitrile by ZrS_1-*y*_S_2-*x*_(15/100) under simulated sunlight irradiation. (H) H_2_O_2_ and benzonitrile evolution rate by the ZrS_3_, ZrSS_2-*x*_(15), and ZrS_1-*y*_S_2-*x*_(15/100) NRs under simulated sunlight irradiation. Reproduced with permission [185]. Copyright 2021, Nature Publishing Group.

The layered crystal structure makes TMTCs a promising candidate for energy storage devices. The NbS_3_ has been adopted as the cathode of magnesium–zinc solid-state batteries, while the 3D interconnected TaS_3_ NWs networks have been used as anode materials for flexible Li-ion batteries (Fig. 8A) [175,176]. Without any protection, the TaS_3_ NWs-base batteries exhibit a decent specific capability of 400 mAh g^−1^, which is better than commercially available graphite material (372 mAh g^−1^). In addition, compared with the initial charge capacity, only 0.1% of capacity is degraded by per cycle, and a high capacity of 60 mAh g^−1^ has been observed after 100 cycles, indicating satisfactory cyclic stability, as shown in Fig. 8B. The excellent properties are derived from the conductive network formed by the 3D interconnected TaS_3_ NWs. A continuous electron path has been formed for fast electron transfer and provides a large electrolyte contact area. More importantly, the structures of 3D TaS_3_ NWs have been retained after 100 cycles, suggesting that the cyclic strain induced by Li insertion/extraction is relieved by the interconnected NWs network. In addition to TaS_3_ NWs, NbSe_3_ NRs have also been employed as anode with the covering of reduced graphene oxide (rGO) to improve the performance of Li-ion batteries [177]. The volume expansion and structural instability of the electrode material during lithium intercalation and deintercalation were largely suppressed, which enhanced the cycling stability and specific capability. Moreover, the discharge capacity was maintained (300 mAh g^−1^) after 250 cycles. Most of the redox activity of battery materials is concentrated on transition metals, while the working mechanism of novel battery electrodes indicated that anionic redox also existed in the working process [178]. The reversible transition between S_2_^2−^ and 2S^2−^ is considered the reason for the high specific capacity of TiS_3_ batteries. The electrons of Li ions are simultaneously transferred to alkali metal atoms and sulfur atoms upon intercalation. This mechanism has also been confirmed by nuclear magnetic resonance (NMR) spectroscopy of natural abundance solid-state ^33^S in NbS_3_ [179].

The DFT calculations show that the valence band mainly depends on the *p*-state of the chalcogen, while the conduction band typically relies on the *d*-state of the transition metal in monolayer MX_3_. Such dependence results in a high-power conversion efficiency of 16% to 18% for MX_3_ thin-film solar cells, such as ZrS_3_ and HfS_3_, which is promising for solar energy storage [1,180]. The 2D vdW hetero-bilayer formed by monolayer ZrS_3_ and monolayer TMDCs exhibits type II band alignment [180]. Specifically, the energy of the conduction band edge of monolayer ZrS_3_ is lower than that of monolayer TMDCs, making them the acceptor and donor in solar energy conversion, respectively, to achieve a synergistic effect. In theory, the power conversion efficiency of the hetero-bilayer solar cells can reach 16%, which paves a new way for efficient solar energy conversion on the nanoscale. The exciton binding energy of ZrS_3_ along the [100] direction is only 0.15 eV, which is beneficial for separating electron–hole pairs. As a result, the collection of photovoltaic current is expected to be along the [100] direction. The TiS_3_ NRs have been used as photoanodes in photoelectrochemical cells, providing a suitable energy band positions for water reduction; i.e., the conduction band position is more negative than the reduction potential of water, and the valence band position is more positive than the oxidation potential of water (Fig. 8C) [181,182].

TiS_3_ has also been widely utilized as electrode material for supercapacitors [183,184]. In the battery-type supercapacitors with asymmetric structure, as displayed in Fig. 8D, the highest capacitance of the TiS_3_-based supercapacitor was 235 F g^−1^ (105 C g^−1^) at 5 mV s^−1^. More importantly, the capacitance was retained (about 91%) after 6,000 galvanostatic charge–discharge cycles (Fig. 8E) [183].

### Catalysts

The structure of TMTC material can also be expressed as M^4+^X^2−^(X_2_^2−^), while all the oxidation state of metal atoms is tetravalent. Unlike TMDCs, TMTCs are more likely to generate X^2−^ and X_2_^2−^ vacancies to form nonstoichiometric TMCs, which is highly desirable for improving catalytic performance [185–187]. Ribbon morphology can be easily formed, and abundant unsaturated X^2−^ ions will be exposed at the edges as reactive active sites [185]. All these characteristics make TMTCs promising photocatalytic and electrocatalytic materials.

In terms of photocatalysis, defective ZrS_3_ nanobelts were used to catalyze the reaction of water and oxygen for synthesis of hydrogen peroxide (H_2_O_2_), and simultaneously oxidize benzylamine to benzonitrile [185]. Under simulated sunlight illumination, the H_2_O_2_ and benzonitrile yields with ZrS_3_ NRs as photocatalysts are 78.1 ± 1.5 and 32.0 ± 1.2 μmol h^−1^, respectively. This is because the conduction band of ZrS_3_ mainly consisted of Zr *d*-orbitals, resulting in a negative potential larger than the reduction potential of O_2_ to H_2_O_2_ [188]. Both experimental and theoretical studies have shown that S_2_^2−^ is beneficial for separating photogenerated carriers (Fig. 8F to H). The negative ion vacancies on the surface of ZrS_3_ NRs are also favorable for transferring holes. Introducing S_2_^2−^ vacancies into ZrS_3_ NRs via high-temperature vacuum annealing treatment further enhances photocatalytic efficiency. The Li-based complex has been utilized for solvothermal treatment to induce more S^2−^ vacancies, improving the electron conduction and hole extraction during the photocatalytic process, and changing benzylamine oxidation kinetics. In addition to ZrS_3_, TiS_3_ has also been adopted as a photocatalyst for pollutant degradation [186,189]. TiS_3_ with a small bandgap enables the absorption of light from the visible to near-infrared region, which enhances the efficient conversion of solar energy. In the degradation experiment of methyl orange dye under simulated sunlight, TiS_3_ showed high catalytic activity compared with other titanium chalcogenides and even higher than TiO_2_. Similar to ZrS_3_, sulfur vacancies can be introduced in TiS_3_ NRs after annealing, which further improves its photocatalytic activity. Moreover, the formation of TiO_2_ passivation layer leads to the TiO_2_/TiS_3_ heterostructure during the photocatalytic process. The heterojunctions and sulfur vacancies help separate photogenerated charges and achieve high-efficiency catalytic reactions. In addition, some reports have shown that the small bandgap of TiS_3_ also provides additional thermocatalytic activity [186].

ZrS_3_ ultrathin nanosheets have also been used as electrocatalysts for efficient oxygen evolution reactions [110]. There are abundant disulfide bonds on the surface of ZrS_3_. Excellent water oxidation activity has been achieved, which includes low onset overpotential of 244 mV and Tafel slope of 45 mV decade^−1^ in an alkaline solution at pH 14. The high oxidation activity can be maintained even under neutral conditions (pH 6.9), which has potential applications for total water splitting. The 2D ultrathin nanosheets also increase the reaction contact area and promote the electrocatalytic process. The TiS_3_ NRs that are obtained by doping TiS_2_ with Nb could be ascribed to the structural transformation from TiS_2_ to TiS_3_ [190]. The TiS_3_ NRs exhibit improved electrocatalytic hydrogen evolution performance.

### Sensors

**Fig. 9.**
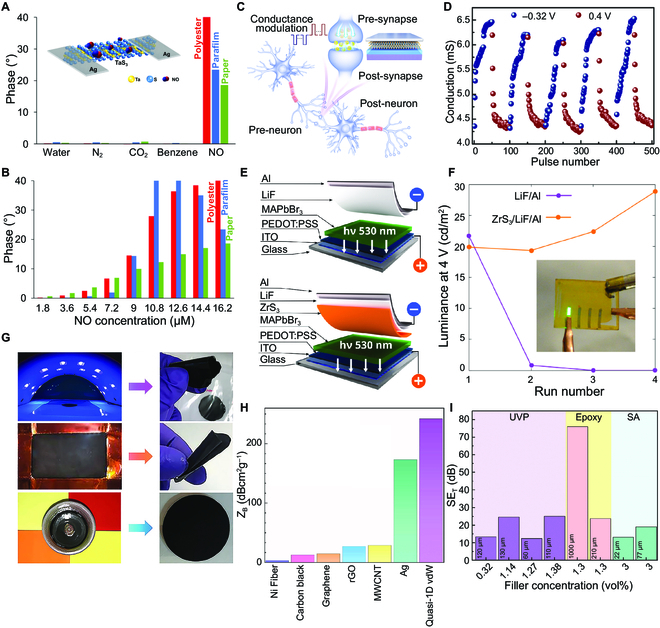
Sensors and other applications of TMTCs. (A) Selectivity of the TaS_3_ nanofiber-based gas sensor. Inset is a schematic of the TaS_3_ nanofiber-based NO gas sensor. (B) Impedance phase responses of the TaS_3_ gas sensor as functions of NO concentration. Reproduced with permission [61]. Copyright 2017, American Chemical Society. (C) Sandwich-like structure of the TiS_3_-based memristor and schematic of modulation of the conductance. (D) Repeated conductance modulation of TiS_3_-based memristor under repeated positive and negative pulses. Reproduced with permission [45]. Copyright 2021, American Chemical Society. (E) Schematic diagram of LED construction with and without ZrS_3_ EIL. (F) Luminance at 4 V for the first 4 voltage sweeps for LEDs in (E). Reproduced with permission [109]. Copyright 2019, American Chemical Society. (G) Flexible films prepared with TaSe_3_ as fillers. (H) Electromagnetic shielding capability shielding characteristics of polymeric composites with different fillers. (I) Total shielding efficiency of all samples tested in the extremely high-frequency band (*f* = 220–320 GHz). Reproduced with permission [196]. Copyright 2021, Wiley-VCH.

Similar to other 2D materials, monolayer or few-layer TMTCs have high specific surface areas. The sulfur vacancies exist at the edge of the TMTC material, which is conducive to the adsorption of gas molecules, making it promising for gas sensors. The adsorption energy of gas molecules on surfaces affects the gas-sensing properties. The selectivity of the TiS_3_ gas sensor has been investigated via the statistical view of surface adsorption and the adsorption strength value, which has been predicted and verified by the programming gas adsorption spectra [191]. It is indicated that five typical adsorption gas molecules (hydrogen, methane, water, oxygen, and ethanol) on the (001) surface of TiS_3_ can be divided into two types. One is the strong adsorption of polar molecules, including ethanol, water, and oxygen, while another is weak adsorption (nonbinding) to nonpolar molecules of hydrogen and methane. This interesting phenomenon is caused by the bipolar interactions between polar molecules and the (001) surface of TiS_3_. The sensing performance of TiS_3_ NRs and graphene–TiS_3_ heterojunctions has been evaluated with the target gases of ethanol, methanol, and acetone at RT [192]. The graphene–TiS_3_ heterojunction sensor exhibits high selectivity to ethanol at RT. The vdW contact between graphene and TiS_3_ effectively reduces the metal-induced defect energy level caused by the interaction between TiS_3_ and Au, resulting in negligible Fermi level pinning (FLP) at the contact. The larger Schottky barrier of the Au-Gr-TiS_3_ sensor is also beneficial for the absorption and desorption of ethanol molecules. TaS_3_ nanofibers have also been utilized as a gas sensor, which exhibits good selectivity against common interfering gases produced during fuel combustion (Fig. 9A and B) [61]. A minimum detection limit of 0.48 ppb for NO has been achieved, which is far below the environmental value of NO_x_ (50 ppb). Moreover, the detection limit is much lower than other reported materials, such as MoS_2_ and TaS_2_. These excellent properties make TMTCs attractive in environmental protection.

### Other applications

Furthermore, some other intriguing applications of TMTCs have been investigated such as fiber lasers, photoelectric memristor, light-emitting diode (LED), and fillers in functional composites. For example, TiS_3_ and ZrTe_3_ are attractive in nonlinear optics, which have been utilized as saturable absorbers to fiber lasers [107,193 ,194]. Ultrashort pulses of 506.5 ns and 1.44 ps can be generated for these optical devices. Liu et al. [45] demonstrated a photoelectric memristor based on TiS_3_ (Fig. 9C and D). Multilevel storage of light with different wavelengths (400 to 808 nm) was realized through the large light absorption range of TiS_3_. The biological synapse function was emulated by the conductance modulation of the TiS_3_-based memristor. Based on this artificial synapse, Pavlov's associative learning behavior was established, proving the potential application of TMTCs in optoelectronic neuromorphic computing systems. The electron injection layer (EIL) of 2D ZrS_3_ has the ability to reduce the turn-on voltage (2.8 V) of LED [109]. The interface between the electrode and the photoactive layer was more stable compared to a LED without ZrS_3_ EIL. The ZrS_3_ thin films in this device are fabricated by the slot-die coating method with optimal ZrS_3_ ink, which was prepared via liquid phase exfoliation from crystals, as displayed in Fig. 9E and F. The introduction of NbSe_3_ nanofibers into base oil can improve the tribological properties and is expected to be used in the industrial lubrication field [195].

The flexible polymer-TaSe_3_ NWs composite films demonstrated superior electromagnetic shielding capability in both X-band (8.2 to 12.4 GHz) and sub-terahertz frequency range (220 to 320 GHz) (Fig. 9G to I) [196,197]. The excellent electromagnetic shielding property has been attributed to the efficient coupling of electromagnetic waves and the TaSe_3_ NWs. The free carriers in quasi-1D TaSe_3_ interact with the electric field of the electromagnetic wave, which has led to the reflection and absorption of electromagnetic energy. The composite film with a TaSe_3_ NW concentration of 4.5 vol% provides a total electromagnetic shielding of about 20 dB in X-band frequency range, which is superior to other conventional composites with metals, CNTs, and graphene. In the sub-terahertz band, the total shielding effect varied from 60 dB to more than 70 dB, which has broad applications in the field of 5G communication and beyond.

## Conclusion and Outlooks

TMTCs exhibit remarkable electrical and physical properties owing to their unique quasi-1D chain structure. They have great potential in the applications of novel electronic, optoelectronic devices, and high-performance integrated logic circuits. We summarize the state-of-the-art achievements of TMTCs, which include crystalline structure, electronic structure, synthesis method, physical properties, and related applications. In recent years, some substantial progresses have been made in TMTC materials. Compared to other 2D materials, the research on TMTCs is still in its early stage. Several issues still need to be solved for the systematic understanding of the properties of TMTCs and the realization of their practical applications.

Compared to 2D materials, TMTCs can be reduced to the 1D atomic scale, making them promising for higher levels of integration in integrated optoelectronic platforms. Controllable synthesis of high-quality, large-scale monolayer or few-layer TMTCs is a crucial and necessary starting point for fabricating high-performance TMTC devices. Currently, the synthesis of TMTCs mainly relies on the CVT method, which suffers from a long reaction cycle and complicated process to fabricate low-dimensional devices. Thus, effective strategies need to be developed. The CVD method is an excellent strategy for the controllable growth of monolayer and few-layer TMDCs. Nonetheless, monolayer TMTCs have not been synthesized by the CVD method. Integrating machine learning (ML) into the CVD process is beneficial for finding out the key parameters in the growth of monolayer TMTCs. In the CVD growth of quasi-1D TMTCs, it is equally critical to suppress the growth perpendicular to the layer direction as to control the growth perpendicular to the 1D chain direction. Compared to 2D materials, the growth conditions of TMTCs are considerably more demanding. Auxiliary feedback from ML can help to achieve more accurate control of thermodynamic and kinetic parameters such as temperature and gas flow rate during CVD reactions, as well as optimization of growth parameters. At the same time, the introduction of ML can also speed up the exploration process and reduce costs. As a quasi-1D structure, studies on single-chain or few-chain TMTCs are meaningful. It is vital to develop methods to isolate and transfer materials with single-chain or countable-chain TMTCs. The top-down exfoliation method is uncontrollable, and it is difficult to obtain a high-quality and stable 1D chain structure. The template-dependent method is an effective strategy, while eliminating the effect of template on material properties is still a challenging problem.

Another problem caused by the limitations of the synthesis technique is that the theoretical calculation results of the physical and electrical properties of TMTCs lack experimental verification. Tensile strain greatly affects the bandgap, mobility, etc. of TMTCs, but experimental demonstrations in these areas are far behind. DFT calculations show that the TMTC material has a high solar energy conversion efficiency (16%), which needs to be verified by experimental results in the future. Compared with TMDCs, the innovative applications of quasi-1D TMTCs are still waiting to be explored. For example, ZrTe_3_, NbSe_3_, and TaS_3_ have been proven to have remarkable CDW property through theoretical and experimental studies. The anticipated application in quantum computing and information processing needs to be implemented. The high breakdown current density (>100 MA cm^−2^) and quasi-1D structural properties make metallic MTe_3_ (i.e., ZrTe_3_) an alternative material for Cu wires. Such materials can become the basis for future nanoelectronics and neural network interconnection technologies. More functional devices based on TMTCs need to be developed, such as bioelectronics, flexible electronics, and other fields. Based on the experience of the development of 2D materials such as graphene, these problems will be overcome in the near future after attracting the attention of a growing number of researchers. It is expected that TMTCs will be a sort of rising star material in the following post-information age.
